# Procalcitonin guided antibiotic therapy of acute exacerbations of asthma: a randomized controlled trial

**DOI:** 10.1186/1471-2334-13-596

**Published:** 2013-12-17

**Authors:** Jianguo Tang, Wei Long, Lei Yan, Yu Zhang, Juan Xie, Gang Lu, Chunhui Yang

**Affiliations:** 1 Department of Trauma-Emergency & Critical Care Medicine, Shanghai Fifth People’s Hospital, Fudan University, Shanghai 200240, PR China; 2 Department of Geriatrics, Shanghai Sixth People’s Hospital, Jiaotong University, Shanghai 200233, PR China; 3 Department of Pulmonary Medicine, Shanghai Fifth People’s Hospital, Fudan University, Shanghai 200240, PR China

**Keywords:** Asthma, Procalcitonin, Antibiotic

## Abstract

**Background:**

This randomized controlled trial aimed to evaluate whether the serum procalcitonin (PCT) level can be utilized to guide the use of antibiotics in the treatment of acute exacerbations of asthma.

**Methods:**

A total of 293 consecutive patients with suspected asthma attacks from February 2005 to July 2010 participated in this study. 225 patients completed the study. Serum PCT levels, and other inflammatory biomarkers of all patients were measured. In addition to the standard treatment, the control group received antibiotics according to the attending physicians’ discretions, while the patients in the PCT group were treated with antibiotics according to serum PCT concentrations. Antibiotics usage was strongly discouraged when the PCT concentration was below 0.1 μg/L; discouraged when the PCT concentration was between 0.1 μg/L and 0.25 μg/L; or encouraged when the PCT concentration was above 0.25 μg/L. The primary endpoint was the determination of antibiotics usage. The second endpoints included the diagnostic accuracy of PCT and other laboratory biomarkers the effectiveness of asthma control, secondary ED visits, hospital re-admissions, repeated needs for steroids or dosage increase, needs for antibiotics, WBC count, PCT levels and FEV1%.

**Results:**

At baseline, two groups were identical regarding clinical, laboratory and symptom score. Probability of the antibiotics usage in the PCT group (46.1%) was lower than that in the control group (74.8%) (χ^2^ = 21.97, *p* < 0.001. RR = 0.561, 95% CI 0.441-0.713). PCT and IL-6 showed good diagnostic significance for bacterial asthma (r = 0.705, *p* = 0.003). The degrees of asthma control in patients were categorized to three levels and were comparable between the two groups at the six weeks follow-up period (χ^2^ = 1.62, *p* = 0.45). There were no significant difference regarding other secondary outcomes (*p* > 0.05).

**Conclusions:**

The serum PCT concentration can be used to effectively determine whether the acute asthma patients have bacterial infections in the respiratory tract, and to guide the use of antibiotics in the treatment of acute asthma exacerbations, which may substantially reduce unnecessary antibiotic use without compromising the therapeutic outcomes.

**Trial registration:**

ICTRP ChiCTR-TRC-12002534

## Background

Acute attacks of asthma are episodes that occur suddenly and may also occur repeatedly over time. The attacks seriously disturb the patient’s daily and work life, which impose great burdens on the family and the society. There are various causes for acute asthma, including allergen provocation, drugs, respiratory viruses, bacterial infections and so on. Since bacterial infection seems to only play a minor role in acute exacerbations of asthma, guidelines for asthma management do not recommend routine use of antibiotics [1]. The majority of patients with acute asthma are treated with a number of conventional treatment measures, including repetitive administration of rapid-acting inhaled bronchodilator, introduction of systemic glucocorticosteroids, oxygen supplementation and so on, to alleviate the symptoms. However, clinical signs and symptoms and laboratory parameters of bacterial infection are often inconclusive, which makes it difficult for physicians to determine accurately in clinical practice whether there is bacterial infection in the respiratory tracts of patients. As a result, most patients are treated with antibiotics, leading to antibiotics abuse and bacterial resistance [2].

Procalcitonin (PCT) is the prehormone of calcitonin. The serum PCT level significantly increases in patients with bacterial infections but not viral infection or other inflammatory diseases, granting the PCT test high potential in discriminating bacterial and non-bacterial infections [3-5]. Therefore, the serum PCT level can guide the use of antibiotics in respiratory tract infections to prevent the abuse of antibiotics in the intensive care unit [6-15]. This study aimed to investigate whether the serum PCT level has diagnostic potential for bacterial asthma attack, and can serve to guide the use of antibiotics in the management of acute exacerbations of asthma.

## Methods

### Setting and study population

From February 2005 to July 2010 in the emergency department of the fifth people’s hospital of Shanghai, 293 suspected acute exacerbation of asthma patients were screened for eligibility to participate in this study. Of these, 265 patients meeting the Bronchial Asthma Guide were eligible and 255 patients completed the study, including 123 male patients and 132 female patients. Patients were eligible for the intervention if they met all the following criteria: 1) ≥ 18 years old; 2) has any, or all, of the following clinical features as defined by the Global Initiative for National Asthma (GINA) Guidelines: dyspnea, wheeze, acute cough, increased work of breathing, increased requirement for beta2-agonist from baseline use, O_2_ saturation <95%, a peak expiratory flow (PEF) at randomization ≤80% of their known best (within the last 12 months) or, in the absence of this information, of their predicted PEF [1]. The exclusion criteria in the study are: 1) treatment with antibiotics within two weeks just before the recruitment; 2) bacterial infection in other parts of body than the respiratory system; 3) chest X-ray confirmed pneumonia; 4) suffering from other chronic respiratory diseases; 5) suffering from severe organ dysfunction. The protocol was approved by the Ethics Committee of the Fudan University. Written informed consent was obtained from all patients or their legally authorized representative.

### Experimental protocol

**Figure 1:**
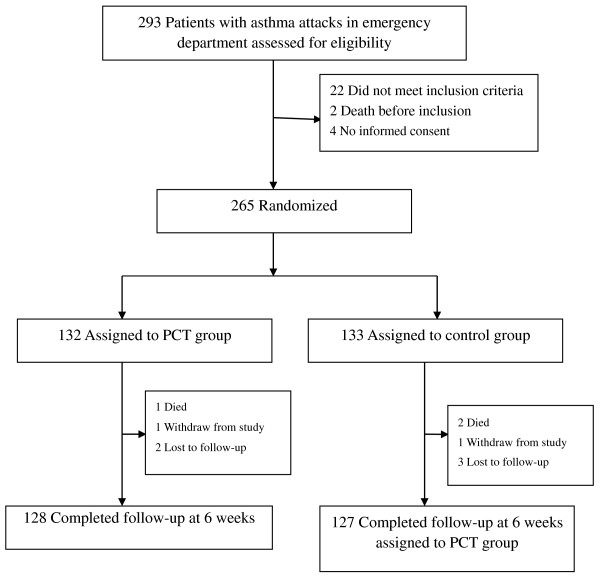
Patient flow chart.

We conducted a randomized, controlled trial. All eligible patients were assigned to either the PCT-guided antibiotics therapy group or the control group, in which the attending physicians decide the use of antibiotics according to usual practice. The flowchart of patients was summarized in Figure 1. Allocation to either intervention was conducted according to computer-generated random numbers produced by an independent statistician. After randomization, an opaque, sealed and sequentially numbered envelope containing the PCT or control protocol was prepared for each subject according to the group. All patients received similar conventional treatment, such as the repetitive administration of rapid-acting inhaled bronchodilator, introduction of systemic glucocorticosteroids and oxygen supplementation. Procalcitonin levels of patients in the PCT group were notified to the attending physician in charge of this group. Patients in the PCT group were treated with antibiotics according to their PCT serum level according the following guidelines: antibiotics treatment was strongly discouraged when serum PCT level was less than 0.1 μg/L; antibiotics treatment was discouraged when serum PCT level was less than 0.25 μg/L; antibiotics treatment was encouraged when serum PCT level was greater than 0.25 μg/L [11]. In contrast, attending physicians responsible for patients in the control group remained unaware of the patients’ procalcitonin concentrations throughout the study. The control group received antibiotics according to the attending physicians’ decision. The patients with PCT < 0.25 ng/ml in the first PCT test would take the PCT test again after six to eight hours. The use of antibiotics would be determined by the second PCT test result.

We appointed an independent investigator team blinded to the group assignment to monitor the adherence to the protocol, the safety and efficacy of the intervention, as well as primary and secondary outcomes during the six-week follow-up period. All patients, laboratory technicians, investigators and research designers were blinded to patient assignments until the data analysis was completed. There were no protocol violations during the study.

### Outcome measures

Our primary endpoint was the use of antibiotics, which was evaluted by the antibiotic prescription rate and the relative risk of antibiotic exposure in patients with acute exacerbations of asthma. The secondary endpoints were patients’ clinical, laboratory, and lung function outcomes at the follow-up visit (6 weeks). During the six-week follow-up period, secondary ED visits, hospital re-admissions, repeated needs for steroids or dosage increase, needs for antibiotics, white blood cell count (WBC), PCT levels and FEV_1_% were assessed.

### Data collection

The serum PCT tests were done using a time-resolved amplified cryptate emission (TRACE) technology assay (Kryptor® PCT, Brahms AG, Hennigsdorf, Germany) with a functional assay sensitivity of 0.06 g/L, about four-fold above the mean normal PCT level. And several laboratory parameters of the patients, including the levels of high sensitivity C-reactive protein (hsCRP), interleukin 6 (IL-6), and tumor necrosis factor α (TNF-α), as well as WBC, were measured and collected. Arterial blood gas analysis was also performed on all patients. The usage of antibiotics in two groups was recorded. Forced expiratory volume in one second (FEV_1_) was collected before and after treatment. The symptom scores of asthma were also recorded. The patients were categorized into mild, moderate, and severe subgroups based on the severity level of their asthma, according to which antibiotics use was analyzed. The patients were re-categorized during the six-week follow-up period to assess the asthma control.

### Statistical analysis

We assumed that 75% of the patients in the control group would use antibiotics and anticipated a 20% decrease in antibiotics usage in the PCT group. The current sample size of 128 patients in the PCT group and 127 in the control group yielded a power of 0.96 to detect difference in usage of antibiotics at 1-sided 5% significance level. Moreover, the power was 1.00 for non-inferiority testing on asthma control at a significance level of 2-sided 0.05, “controlled” percentage of 75% in both PCT and control groups and a non-inferiority limit (d) of 65%.

Discrete variables are expressed as counts (percentages) and continuous variables as mean ± SD. Comparability of groups was analyzed by χ^2^ test, two-sampled t test, Mann–Whitney U test, as appropriate. Correlation analyses of the level of serum PCT and levels of hsCRP, IL-6, TNF-α and WBC were performed using Spearman rank. A *p* value less than 0.05 was defined as significant. Data were analyzed using statistical software (Statistical Package for Social Sciences, version 14 for Windows; SPSS; Chicago IL).

## Results

The patients were aged between 19 and 78 years old and the average age was 53 ± 15 years old. Detailed baseline characteristics of the studied population are presented in Table 1.

**Table 1:** Baseline characteristics of the two groups

**Characteristic**	**PCT group (n = 128)**	**Control group (n = 127)**
Median age, yr	54±14	55±15
Mean Asthma history, yr	25±17	27±18
Gender, no. (%)		
Male	64 (50.0)	59 (46.5)
Female	64 (50.0)	68 (53.5)
HR, times/min	95±24	97±26
RR, times/min	24±12	25±10
pH	7.39±0.06	7.38±0.05
SaO_2_, %*	94±5	95±3
PaO_2_, mmHg*	79±19	75±17
PaCO_2_, mmHg*	44±12	42±10
WBC, ×10^9^/L	12.1±4.6	11.9±5.2
hsCRP, mg/L	8.2 (4.5 ~ 15.7)	6.9 (5.1 ~ 17.6)
PCT, ng/ml	0.137 (0.068 ~ 0.252)	0.119 (0.057 ~ 0.267)
IL-6, pg/ml	14.5 (8.3 ~ 26.1)	17.3 (6.7 ~ 31.4)
TNF-α, pg/ml	27.5 (13.4 ~ 49.5)	32.4 (15.7 ~ 52.1)
Severity of asthma		
Mild	47 (36.7)	50 (39.4)
Moderate	54 (42.2)	52 (40.9)
Severe	17 (13.3)	17 (13.4)
Critical	10 (7.8)	8 (6.3)
FEV_1_, %	57.8±17.2	54.3±16.7
Mean symptom score	4.42±0.85	4.35±0.79

Note: Data are presented as No. (%), M (Q_L_ ~ Q_U_) or mean ± SD; * = the value after oxygen.

The FEV_1_ score of both groups was significantly improved after treatment compared with that before treatment. Thirty-two (25.0%) patients in PCT group received corticosteroids therapy, while 36 (28.3%) patients received corticosteroids therapy in the control group (χ ^2^ = 0.13, *p* = 0.714). Eight (6.3%) patients in PCT group and nine (7.1%) patients in the control group (χ ^2^ = 0.05, *p* = 0.821) received mechanical ventilation treatment.

Table 2 showed the details of the levels of inflammatory parameters in patients. The patients with severe-critical asthma had higher serum PCT levels and IL-6 levels compared with those with mild-moderate asthma (*p* < 0.05). Other inflammatory parameters that we tested showed no significant difference between patients with mild-moderate asthma and severe-critical asthma. Spearman analysis showed that the serum PCT level didn’t correlate with the level of any laboratory biomarkers except for IL-6 (r = 0.705, *p* = 0.003).

**Table 2:** Comparison of inflammatory variables

**Level**	**Inflammatory parameters**				
	**PCT(ng/ml)**	**hsCRP(mg/L)**	**IL-6(pg/ml)**	**WBC(×10**^**9**^**/L)**	**TNF-α(pg/ml)**
Mild to Moderate (n = 203)	0.044 (0.027 ~ 0.241)	6.7 (3.8 ~ 13.5)	8.1 (4.5 ~ 13.4)	11.7±4.2	29.3 (12.5 ~ 46.2)
Severe to Critical (n = 52)	0.263 (0.158 ~ 0.482)	7.4 (4.9 ~ 16.4)	21.2 (13.7 ~ 48.1)	12.1±3.8	31.7 (14.1 ~ 50.6)
Statistic value	*Z* = −2.324	*Z* = −0.538	*Z* = −3.217	*t* =0.013	*t* = −0.615
*p* value	0.041	0.557	0.036	0.989	0.805

Note: Data are presented as M (Q_L_ ~ Q_U_) or mean ± SD.

As shown in Table 3, only 46.1% of the patients in PCT group received antibiotics therapy, which was much lower compared with 74.8% in the control group (χ^2^ = 21.97, *p* = 0.000, RR = 0.561, 95% CI 0.441-0.713). Table 3 also showed that the usage of antibiotics in the PCT group patients with mild and moderate asthma were significantly lower than that in the control group (*p* <0.01). There was no significant statistical difference in the usage of antibiotics between patients with severe and critical asthma in the two groups.

**Table 3:** Comparison of the usage of antibiotics

	**Usage of antibiotics, (%)**		**χ**^**2**^	***p *** **value**
	**PCT group**	**Control group**		
Mild	16/47 (34.0)	37/50 (74.0)	15.61	<0.01
Moderate	21/54 (38.9)	36/52 (69.2)	9.81	<0.01
Severe	13/17 (76.5)	14/17 (82.4)	0.18	= 0.671
Critical	9/10 (90.0)	8/8 (100.0)	0.85	= 0.357
Total	59/128 (46.1)	95/127 (74.8)	21.97	<0.01

The asthma control categorized to three levels was comparable between two groups during the six-week follow-up period (χ^2^ =1.62, *p* = 0.45) (Table 4). There were no significant difference regarding other secondary outcomes such as secondary ED visits, hospital re-admissions, repeated needs for steroids or dosage increase, needs for antibiotics, WBC, PCT levels and FEV1% between two groups (p > 0.05).

**Table 4:** Comparison of the levels of asthma control and the follow-up outcomes between two groups

	**PCT group**	**Control group**	
**Levels of asthma control**			
Controlled	102	97	
Partly controlled	20	19	
Uncontrolled	6	11	
Total	128	127	χ^2^ = 1.62 *p* > 0.05
**Other follow-up outcomes**			
Secondary ED visits	8	13	χ^2^ = 1.43 *p* > 0.05
Hospital re-admissions	5	8	χ^2^ = 0.75 *p* > 0.05
Repeated needs for steroids or dosage increase*	6	9	χ^2^ = 0.66 *p* >0.05
Needs for antibiotics	5	9	χ^2^ = 1.24 *p* > 0.05
WBC, ×10^9^/L	8.68±2.47.2	8.32±2.93	*t* =1.08 *p* = 0.283
PCT, ng/ml	0.033 (0.024 ~ 0.039)	0.036 (0.027 ~ 0.040)	*Z* = 1.29 *p* = 0.071
FEV_1_%	68.20±10.21	70.17±8.89	*t* = −1.64 *p* = 0.103

*Means IV, PO or Inhalation; Data are presented as M (Q_L_ ~ Q_U_) or mean ± SD.

## Discussion

This study showed that PCT test could help physicians to determine whether patients were suffering from respiratory tract bacterial infections. As a result, PCT test increases the safety level of the treatment strategies of acute exacerbations of asthma via avoiding antibiotics abuse. The study published by Briel et al. was not particularly convincing since only nine asthma patients were included [9]. Thus far this is the only clinical study focusing on evaluating the PCT test in its effectiveness to guide antibiotics usage in treating acute exacerbations of asthma.

PCT, a protein of 116 amino acids with a molecular weight of 13 kDa, was discovered 25 years ago as a prohormone of calcitonin produced by C-cells of the thyroid gland [16]. Under normal physiological conditions, PCT is stable in human body [17]. PCT is intracellularly cleaved by proteolytic enzymes to generate the active hormone. Circulating levels of PCT in healthy subjects are below detection limit. It was found in 1993 that the serum PCT level is elevated in patients with bacterial infection [18]. Since then PCT has become an important protein in the detection and differential diagnostics of inflammatory states [3-5]. In microbial infections and in various forms of inflammation, circulating levels of PCT increase to several thousand-fold of its normal level [18,19]. In this case, PCT is produced by other tissues, especially adherent monocytes and macrophage-activated adipocytes [19]. This increase correlates significantly with the severity of the condition and with mortality. However, PCT level does not increase markedly in patients with autoimmune inflammation or virus infection [3-5,7]. PCT is released within four hours in the initial stages of infection, and peaks in eight hours. Then PCT level begins to decrease when the infection is under control. The half-life of PCT is between 20 and 24 hours [17,20]. The serum PCT level is interfered by hormone levels and can easily be detected in a short time [21]. Small doses of intravenous lipid polysaccharide in healthy volunteers could induce the production of PCT [21]. The serum PCT can be detected after two hours and the PCT level increased rapidly in the following six to eight hours [21]. The PCT level reaches the peak l2 to 48 hours later and returns to normal in two to three days [22]. Respiratory tract infections induce the acute attacks of asthma. Bacterial infection seems to just play a minor role in acute exacerbations of asthma, while virus infection plays a major role [1,23,24]. So many patients with acute asthma don’t need antibiotics. Antibiotic therapy benefits those patients with bacterial infection by alleviating respiratory tract spasm and shortening the duration of acute exacerbation, while it harms patients without bacterial infection by worsening their condition and increasing the chance of selective bacterial resistance [2,23]. The physicians often determine whether patients suffer from respiratory tract bacterial infections on the basis of sputum characteristics, body temperature, WBC, CRP and other indicators. However, clinical signs and symptoms of bacterial infection and other non-bacterial infections are often indistinguishable, making it difficult for physicians to determine accurately in clinical practice whether there is a bacterial infection in respiratory tract of patients [25]. In general, bacterial culture is much accurate for diagnosis. However, bacterial culture takes too long time and it is difficult to obtain accurate information on etiology from asthma patients. Therefore bacterial culture is not suitable for the emergent treatment of asthma. And the positive rate of bacterial culture was limited by the hospital medical standards, medical technicians operating experience and other factors, which makes bacterial culture a less effective measure. When there is no effective approach for physicians to determine accurately whether there is a bacterial infection, physicians are more likely to use antibiotic therapy for most acute asthma patients, leading to antibiotics abuse and subsequent bacterial resistance [2]. Recent studies showed that serum PCT level has higher sensitivity in determining whether bacterial infection exist in community acquired pneumonia, ventilator associated pneumonia and acute exacerbation of chronic obstructive pulmonary disease and can guide the usage of antibiotics [6,8,26]. The serum PCT level is also an early diagnostic index and can identify sepsis from systemic inflammatory response syndrome (SIRS) [3,7,14]. PCT test can objectively determine the severity of sepsis and be closely related to the severity of multiple organ dysfunction syndrome (MODS) [3,7,14]. Physicians can judge the direction of progression and evaluate the therapeutic effectiveness by continuously monitoring the serum PCT level [27,28].

In this study, we treated the patients with antibiotics according to PCT serum level. Antibiotics usage was strongly discouraged when the serum PCT concentration was below 0.1 μg/L; antibiotics usage was discouraged when the serum PCT concentration was between 0.1 μg/L and 0.25 μg/L; and antibiotics usage was encouraged when the serum PCT concentration was greater than 0.25 μg/L. The results showed that many patients in the PCT group did not need antibiotics. The antibiotic usage rate was lower than that of the control group and the outcome of treatment was not significantly affected, especially for patients with mild to moderate asthma. Patients with mild to moderate asthma in the control group did not need antibiotics, which implicated that PCT could play an important role in guiding the use of antibiotics. Although there was no significant statistical difference in the rate of antibiotics usage in patients with severe to critical asthma between the two groups, the trend of antibiotics usage in PCT group deceased in comparison with the control group. Accordingly, we believe that PCT is a serum marker for the appropriate selection of antibiotics in the treatment of acute attack of asthma. PCT can be quickly tested to help determine whether there is bacterial infection and whether antibiotics are needed for the emergency treatment of patients.

In this study, the serum PCT level and IL-6 level were correlated, while there is no correlation between the serum PCT level and levels of other inflammatory indicators, including hsCRP, TNF-α, and WBC. Cellular IL-6 level rises sharply after exposure to bacterial toxins, falls rapidly as time elapses and has a longer half-life than TNF-α [29,30]. As a critical mediator of survival following pulmonary infection and sepsis, it protects patients from death by augmenting neutrophil killing of bacteria, enhancing the release of adrenaline and cortisol, as well as selectively activating the coagulation system [31,32]. IL-6 has the best discriminative power in sepsis and non-infectious SIRS, with sensitivity above 80% and specificity above 70%, which is higher than conventional inflammatory markers including CRP and TNF-α [33]. There are limitations in the sensitivity and specificity of hsCRP, TNF-α, and WBC in judging whether patients with asthma are also suffering from bacterial infection. Both PCT and IL-6 levels of patients with severe to critical asthma in the PCT group were higher than that of patients with mild to moderate asthma in the same group. It implicated that patients with severe to critical asthma may be more prone to bacterial infection than patients with mild to moderate asthma. Zhang et al. found that bacterial colonization of the lower airways was common in patients with chronic severe asthma and was linked to the duration of asthma and the exacerbations in past years [34,35]. Therefore, PCT test prevents antibiotics usage mostly in patients with mild to moderate asthma but without bacterial infection, while most patients with severe to critical asthma do suffer from bacterial infection and need to be treated with proper antibiotics.

## Conclusion

In summary, this study showed the PCT test can be used accurately and effectively to determine whether the acute asthma patients have bacterial infections in respiratory tract, and to guide the use of antibiotics in the treatment of acute asthma. So PCT test has great value in reducing unnecessary antibiotics use and in preventing the occurrences of antibiotics resistance in the treatment of acute asthma, particularly in countries where patients are suffering from the abuse of antibiotics. Our study has several limitations. First, we included a relatively small number of patients. However, for the 128 patients in PCT group and 127 in control group, the power reached 0.99 with 1-sided 5% significance level to detect the difference in usage of antibiotics and was 0.88 for non-inferiority testing with a non-inferiority limit (d) of 65% on asthma control. Second, our results should not be generalized to other settings such as hospital-acquired pneumonia and chronic obstructive pulmonary disease. Third, we did not conduct a formal cost-benefit study. Larger clinical trials are needed to explore the overall clinical and economic impact of the reduction of exposure to antibiotics in asthma patients.

## Competing interests

The authors declare that they have no competing interests.

## Authors’ contributions

LW carried out the investigation, participated in study design and drafted the manuscript. YL and ZY carried out the investigation, participated in the laboratory measuring. XJ participated in the study design, carried out the investigation. LG participated in the design of the study and performed the statistical analysis. YCH carried out the investigation, participated in the laboratory measuring. TJG conceived the idea of the study, and participated in its design and coordination and helped to draft the manuscript. All authors read and approved the final manuscript.

## Authors’ information

Co-first author Wei Long.

## Pre-publication history

The pre-publication history for this paper can be accessed here:

http://www.biomedcentral.com/1471-2334/13/596/prepub
